# Prevalence of myocardial injury in patients after acute ischaemic stroke according to standard criteria

**DOI:** 10.1093/eurheartjsupp/suad104

**Published:** 2023-05-24

**Authors:** Michal Mihalovic, Petr Mikulenka, Hana Línková, Marek Neuberg, Ivana Štětkářová, Tomáš Peisker, David Lauer, Petr Tousek

**Affiliations:** Cardiocenter, Charles University-Third Faculty of Medicine, Ruská 87, 100 00 Prague, Czech Republic; Department of Neurology, Third Faculty of Medicine, University Hospital Kralovske Vinohrady, Charles University, Ruská 87, 100 00 Prague, Czech Republic; Cardiocenter, Charles University-Third Faculty of Medicine, Ruská 87, 100 00 Prague, Czech Republic; Medtronic Czechia, Partner of INTERCARDIS, Prosecká 852/66, 190 00 Prague, Czech Republic; Department of Neurology, Third Faculty of Medicine, University Hospital Kralovske Vinohrady, Charles University, Ruská 87, 100 00 Prague, Czech Republic; Department of Neurology, Third Faculty of Medicine, University Hospital Kralovske Vinohrady, Charles University, Ruská 87, 100 00 Prague, Czech Republic; Department of Neurology, Third Faculty of Medicine, University Hospital Kralovske Vinohrady, Charles University, Ruská 87, 100 00 Prague, Czech Republic; Cardiocenter, Charles University-Third Faculty of Medicine, Ruská 87, 100 00 Prague, Czech Republic

**Keywords:** Stroke, Troponin, Mortality, Cardiovascular disease, Acute ischaemic stroke, Myocardial injury

## Abstract

This study examined the prevalence of acute and chronic myocardial injury according to standard criteria in patients after acute ischaemic stroke (AIS) and its relation to stroke severity and short-term prognosis. Between August 2020 and August 2022, 217 consecutive patients with AIS were enrolled. Plasma levels of high-sensitive cardiac troponin I (hs-cTnI) were measured in blood samples obtained at the time of admission and 24 and 48 h later. The patients were divided into three groups according to the Fourth Universal Definition of Myocardial Infarction: no injury, chronic injury, and acute injury. Twelve-lead ECGs were obtained at the time of admission, 24 and 48 h later, and on the day of hospital discharge. A standard echocardiographic examination was performed within the first 7 days of hospitalization in patients with suspected abnormalities of left ventricular function and regional wall motion. Demographic characteristics, clinical data, functional outcomes, and all-cause mortality were compared between the three groups. The National Institutes of Health Stroke Scale (NIHSS) at the time of admission and the modified Rankin Scale (mRS) 90 days following hospital discharge were used to assess stroke severity and outcome. Elevated hs-cTnI levels were measured in 59 patients (27.2%): 34 patients (15.7%) had acute myocardial injury and 25 patients (11.5%) had chronic myocardial injury within the acute phase after ischaemic stroke. An unfavourable outcome, evaluated based on the mRS at 90 days, was associated with both acute and chronic myocardial injury. Myocardial injury was also strongly associated with all-cause death, with the strongest association in patients with acute myocardial injury, at 30 days and at 90 days. Kaplan–Meier survival curves showed that all-cause mortality was significantly higher in patients with acute and chronic myocardial injury than in patients without myocardial injury (*P* < 0.001). Stroke severity, evaluated with the NIHSS, was also associated with acute and chronic myocardial injury. A comparison of the ECG findings between patients with and without myocardial injury showed a higher occurrence in the former of T-wave inversion, ST segment depression, and QTc prolongation. In echocardiographic analysis, a new abnormality in regional wall motion of the left ventricle was identified in six patients. Chronic and acute myocardial injury with hs-cTnI elevation after AIS are associated with stroke severity, unfavourable functional outcome, and short-term mortality.

## Introduction

Stroke is one of the most common causes of death and a main cause of disability worldwide, resulting in a significant socio-economic burden. The pathological disturbances that occur in stroke can lead to autonomic dysfunction, systemic inflammation, and changes in cardiomyocyte metabolism.^1–3^ Myocardial injury and cardiac complications after stroke may result in serious complications, including heart failure, arrhythmias, and death.^4,5^

Current guidelines from the United States recommend measuring cardiac biomarkers, including high-sensitive cardiac troponin I (hs-cTnI),^6,7^ in patients admitted for acute ischaemic stroke (AIS) with recommendation Class I.^8^ The Fourth Universal Definition of Myocardial Infarction defines several changes, including troponin elevation, and distinguishes between myocardial infarction and myocardial injury.^9–11^ However, the guidelines do not specify the management process in asymptomatic patients with acute stroke and an elevated troponin level, which complicates the diagnosis of acute coronary syndrome and therapeutic decision-making. Myocardial injury after stroke occurs even in the absence of obstructive coronary artery disease and is also characteristic of cardiac conditions such as heart failure, pericarditis, myocarditis, atrial fibrillation, and tachycardia.^12^

Several studies have reported an association between troponin elevation and all-cause death and functional disability,^13–15^ but these relied solely on a single troponin measurement and therefore could not distinguish between patients with acute vs. chronic myocardial injury. Thus, in this study, we examined the dynamic changes in hs-cTnI in AIS patients with acute and chronic myocardial injury, the prevalence of these conditions, patient outcomes, and associations with stroke severity.

## Materials and methods

### Study design and patients

The study population consisted of consecutively enrolled AIS patients seen at the Department of Neurology, University Hospital Kralovske Vinohrady, Prague, between August 2020 and August 2022. Inclusion criteria were AIS diagnosed based on clinical and results of non-contrast CT of the head, supplemented by CT angiography or magnetic resonance imaging findings. Clinical data and other predictor variables (demographics, haemodynamics, and blood results) were obtained. Demographic and patient history data were collected, including age, sex, smoking, previous stroke, or transient ischaemic attack, hypertension, diabetes mellitus, renal impairment (plasma creatinine > 134 mmol/L), and cardiac diseases (coronary artery disease, heart failure, atrial fibrillation). A standard neurological examination was performed, and stroke severity was assessed at the time of admission using the National Institutes of Health Stroke Scale (NIHSS): no stroke symptoms (0), minor (1–4), moderate (5–15), moderate-to-severe (16–20), and severe (21–42). Functional outcomes were evaluated using the modified Rankin Scale (mRS), including death within the first 90 days, based on the following categories: no symptoms (0), no disability despite symptoms (1), slight disability (2), moderate (3), moderate-to-severe (4), severe (5), and death (6). Exclusion criteria at admission included a history of acute myocardial infarction, severe valve disease, known heart failure with reduced ejection fraction, and patients < 18 years. The study was approved by the institutional Ethics Committee, and written informed content was obtained from each patient. The study protocol conformed with the ethical guidelines of the 1975 Declaration of Helsinki.

### Laboratory analysis

Venous blood samples were obtained from patients at the time of admission, and 24 ± 12 and 48 ± 12 h later, at which time the hs-cTnI level was measured (cut-off at our hospital: 53 ng/L for men and 34 ng/mL for women). Patients after AIS were divided into three groups according to the Fourth Universal Definition of Myocardial Infarction: no injury, no measurable hs-cTnI; acute injury, hs-cTnI values above the 99th percentile of the upper reference limit (URL) and an increase and/or decrease in hs-cTnI values > 20%; and chronic injury, hs-cTnI level above the URL but without subsequent change > 20%. Blood values (e.g. haemoglobin level, leukocyte count, and platelet count) were obtained and standard biochemistry tests (potassium, creatinine, and C-reactive protein levels) were performed within the same timeframe.

### Electrocardiogram and echocardiographic examination

Twelve-lead ECGs were obtained at the time of admission, 24 ± 12 h later, 48 ± 12 h later, and at the time of patient discharge. The data were analysed by one cardiologist who was blinded to the patient’s data. The following changes were recorded: atrial fibrillation, atrial flutter, sinus tachycardia (heart rate > 100), sinus bradycardia (heart rate < 60), ventricular tachycardia (more than three beats of ventricular origin), ectopic ventricular beats, ST elevation, ST depression, isoform T-wave, inverted T-wave, U-wave, QTc > 0.45 s for men and >0.46 s for women, and first-, second-, and third-degree atrioventricular block. A standard echocardiographic examination was performed within the first 7 days of hospitalization in patients with suspected left ventricular function and abnormalities of regional wall motion.

### Statistical analysis

Categorical variables were recorded as frequencies or counts (percentages). Continuous data were tested for a normal distribution using the Kolmogorov–Smirnov test. For continuous variables, parameters that followed a normal distribution were analysed using Student’s *t*-test and are reported as the mean ± standard deviation. A chi-square test or Fisher’s exact test was used to detect differences between categorical variables. Univariate and multivariate logistic regression models were used to evaluate the factors influencing an elevated hs-cTnI. Survival was estimated using the Kaplan–Meier method and the results were compared using the log-rank test. The results were considered statistically significant at a *P* value < 0.05.

## Results

### Patients

Among the 217 consecutively enrolled patients, 118 were men. The mean age was 70.3 ± 12.5 years. Reperfusion therapy was administered to 73% of the patients (65 patients underwent mechanical thrombectomy; 146 patients received intravenous thrombolysis). *Table 1* summarises the baseline characteristics of the patients. Fifty-nine patients had hs-cTnI levels above the reference value (>53 ng/mL men, > 34 ng/mL women, 27.2%). Patients with hs-cTnI elevation were older (76.4 ± 12.6 vs. 68.2 ± 9.9, *P* < 0.001). The most common comorbidities included arterial hypertension, dyslipidemia, type 2 diabetes, and atrial fibrillation. Patient comorbidities, such as diabetes, coronary artery disease, atrial fibrillation, and renal insufficiency, were more frequent in the group with elevated levels of hs-cTnI. The majority of patients were admitted within 12 h of symptom onset, with 77.8% in the group without hs-cTnI elevation presenting with minor-to-moderate stroke (NIHSS 1–15) compared with 61% in the group with hs-cTnI elevation. Among patients who presented with moderate-to-severe or severe stroke (NIHSS 16–42), the respective values were 14 and 38% (*P* = 0.001). Echocardiographic examination within 7 days of hospitalization was performed in 155 patients (71.4%); new abnormalities in regional wall motion of the left ventricle (LV) were determined in 6 patients.

**Table 1:** Baseline characteristics of 217 included patients with acute ischaemic stroke

	Injury, *n* = 59	No injury, *n* = 158	*P* value
Baseline characteristics			
ȃAge, Mean year (SD)	76.4 ± 12.6	68.2 ± 9.9	<0.001
ȃMale, *n* (%)	24 (40.7)	94 (59.5)	0.015
ȃArterial hypertension, *n* (%)	46 (78)	123 (77.8)	1
ȃSmoking, *n* (%)	22 (37.3)	63 (39.9)	0.75
ȃDyslipidemia, *n* (%)	34 (57.6)	95 (60.1)	0.76
ȃDiabetes mellitus, *n* (%)	22 (37.3)	32 (20.3)	0.013
ȃIschaemic heart disease, *n* (%)	7 (11.9)	10 (6.3)	0.61
ȃHistory of stroke/TIA, *n* (%)	7 (11.9)	12 (7.6)	0.42
ȃAtrial fibrillation, *n* (%)	16 (27.1)	29 (18.4)	0.19
ȃRenal insufficiency, *n* (%)	4 (6.8)	4 (2.5)	0.22
ȃHistory of myocardial infarction, *n* (%)	3 (5.1)	5 (3.2)	0.45
Chronic medication			
ȃAntiaggregation	19 (32.2)	35 (22.2)	0.16
ȃAnticoagulation	6 (10.2)	20 (12.7)	0.81
ȃBetablocker	22 (37.3)	42 (26.6)	0.13
ȃACE-I/Sartan	28 (47.5)	68 (43)	0.65
Assessments			
ȃSymptom duration			
ȃȃ< 4.5 h, *n* (%)	50 (84.7)	125 (79.1)	0.44
ȃȃ< 12 h, *n* (%)	56 (94.9)	152 (96.2)	0.7
ȃȃWake-up	4 (6.8)	21 (13.3)	0.24
ȃNIHSS			0.001
ȃȃ0 (No stroke symptoms)	0 (0)	0 (0)	
ȃȃ1–4 (Minor)	7 (11.9)	53 (33.5)	
ȃȃ5–15 (Moderate)	29 (49.1)	78 (49.4)	
ȃȃ16–20 (Moderate-to-severe)	16 (27.1)	24 (15.2)	
ȃȃ21–42 (Severe)	7 (11.9)	3 (1.9)	
ȃmRS 90 days			<0.001
ȃȃ0 (No symptoms) (%)	12 (20.3)	60 (37.9)	
ȃȃ1 (No disability despite symptoms) (%)	4 (6.8)	35 (22.2)	
ȃȃ2 (Slight disability) (%)	7 (11.8)	14 (8.9)	
ȃȃ3 (Moderate disability) (%)	4 (6.8)	17 (10.7)	
ȃȃ4 (Moderate severe disability) (%)	3 (5.1)	10 (6.3)	
ȃȃ5 (Severe disability) (%)	5 (8.5)	8 (5.1)	
ȃȃ6 (Dead) (%)	24 (40.7)	14 (8.9)	
Mortality			
ȃHospitalization, *n* (%)	11 (18.6)	8 (5.1)	0005
ȃ90 days, *n* (%)	24 (40.7)	14 (8.9)	<0001

AIS, acute ischaemic stroke; ICH, intracerebral haemorrhage; mRS, modified Rankin Scale; NIHSS, National Institutes of Health Stroke Scale; TIA, transient ischaemic attack.

### Myocardial injury in patients after acute ischaemic stroke

**Figure 1:**
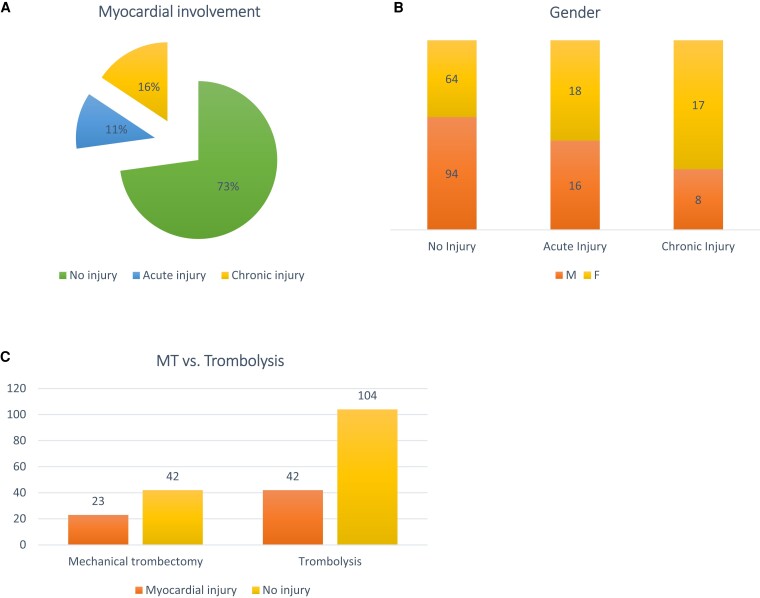
Myocardial injury in patients after acute ischaemic stroke: (*A*) prevalence of acute, chronic and no myocardial injury after acute ischaemic stroke; (*B*) gender differences in patients after acute ischaemic stroke with and without myocardial injury; (*C*) different treatment strategies and prevalence of myocardial injury in patients after acute ischaemic stroke.

In the study population, 158 patients (73%) presented with no injury, 34 (15.7%) had acute myocardial injury, and 25 (11.5%) showed chronic myocardial injury within the acute phase after stroke (*Figure 1A*). The proportion of female patients in both the acute and chronic myocardial injury groups was significantly higher than that of men (*P* = 0.015) (*Figure 1B*). An elevated hs-cTnI level was measured in 35.4% of patients after mechanical thrombectomy and 28.8% of patients after intravenous thrombolysis, but the association between treatment strategy and an elevated hs-cTnI level was not significant (*P* = 0.34) (*Figure 1C*).

In logistic regression, variables with a statistically significant association with hs-cTnI elevation included C-reactive protein > 10 mg/mL [*P* = 0.048, odds ratio (OR) 2.657, 95% confidence interval (CI) 1.009–6.996], type 2 diabetes (*P* = 0.0025, OR 4.809, 95% CI 1.738–13.308), NIHSS > 16 (*P* = 0.013, OR 3.686, 95% CI 1.315–10.335), and age > 75 (*P* = 0.02, OR 3.198, 95% CI 1.166–8.773).

### Associations between hs-cTnI and stroke severity, worse outcome, and death in acute ischaemic stroke patients

**Figure 2:**
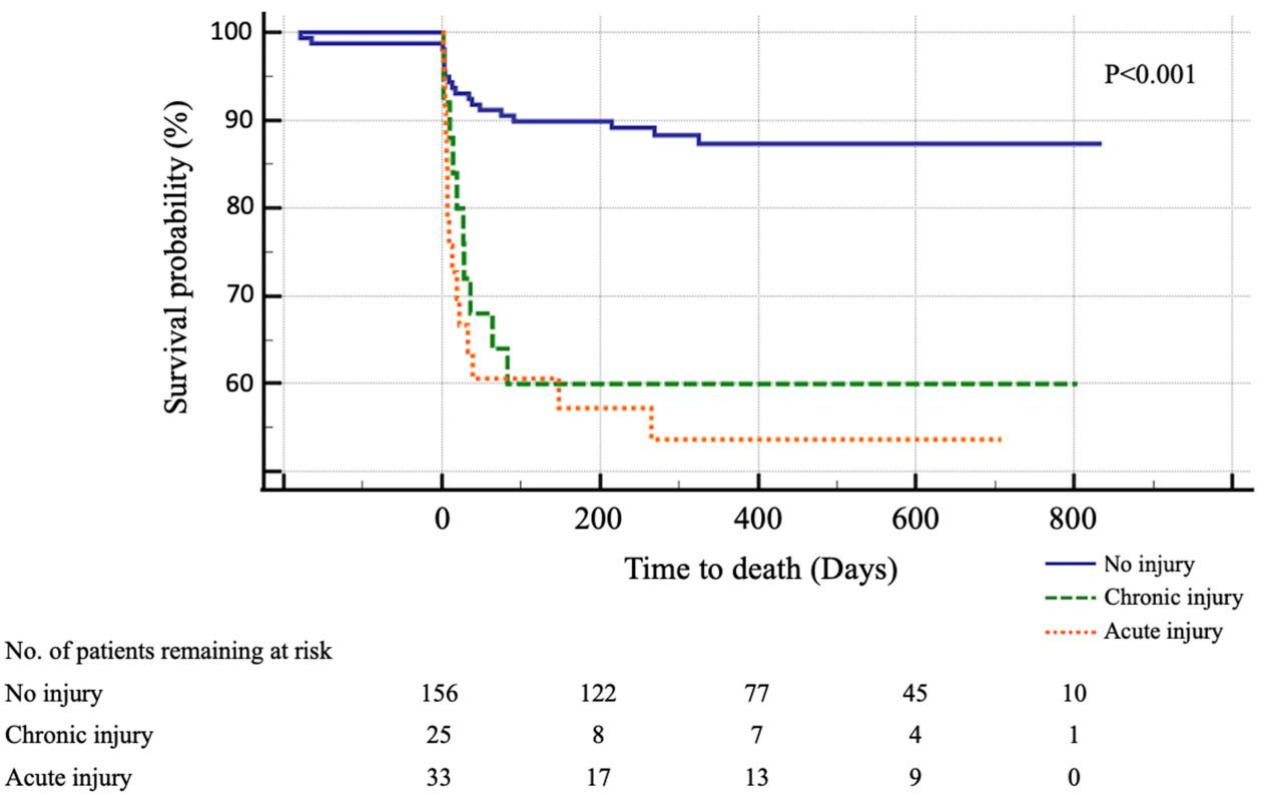
Kaplan–Meier survival curves comparing no injury, chronic injury, and acute injury. The number of patients at risk at baseline for each cut point is shown at the bottom of the graph.

In univariate analyses, chronic injury was associated with 30-day (*P* = 0.006) and 90-day (*P* = 0.001) mortality. The association between acute injury and mortality was even stronger both after 30 days (*P* < 0.001) and after 90 days (*P* < 0.001). At 90 days, patients with an increased hs-cTnI level were at higher risk for an unfavourable outcome (mRS 90 days ≥ 4), in both the chronic (*P* = 0.004) and the acute (*P* = 0.011) myocardial injury groups. Stroke severity was associated with chronic (*P* = 0.05) as well as acute (*P* = 0.02) myocardial injury (*Table 2*A, B). The Kaplan–Meier survival analysis showed a significantly higher all-cause mortality among patients with acute and chronic myocardial injury than without (*P* < 0.001) (*Figure 2*).

**Table 2:** Severity and prognosis in patients with and without myocardial injury

(A)	No injury, *n* = 158	Chronic injury, *n* = 25	Unadjusted OR (95% CI)	*P* value
Death				
ȃ30 days	10	7	4.424 (1.5419, 12.6931)	0.006
ȃ90 days	14	10	4.514 (1.8087, 11.2669)	0.001
Unfavourable outcome				
ȃmRS 90 days ≥4	49	17	2.963 (1.4072, 6.2366)	0.004
Stroke severity				
ȃNIHSS ≥ 16	27	10	2.341 (1.0112, 5.4182)	0.05

(B)	No injury, *n* = 158	Acute injury, *n* = 34	Unadjusted OR (95% CI)	*P* value
Death				
ȃ30 days	10	14	6.506 (2.666, 15.876)	<0.001
ȃ90 days	14	14	4.647 (2.029, 10.639)	<0.001
Unfavourable outcome				
ȃmRS 90days ≥4	49	19	2.469 (1.232, 4.948)	0.011
Stroke severity				
ȃNIHSS ≥ 16	27	14	2.409 (1.145, 5.072)	0.02

(A) Severity and prognosis in patients with chronic myocardial injury compared with no injury. (B) Severity and prognosis in patients with acute myocardial injury compared with no injury.

### ECG and echocardiographic analysis

The most common ECG changes included T-wave inversion (21.2%), ST depression (15.2%), QTc prolongation (13.4%), and a flat T-wave (12%). The association between myocardial injury and ECG changes is summarised in *Table 3*. Patients with myocardial injury had a higher occurrence of T-wave inversion (*P* < 0.001), ST segment depression (*P* < 0.001), and QTc prolongation (*P* = 0.001).

**Table 3:** Relationship between ECG changes, arrhythmias, and myocardial injury in acute ischaemic stroke

	No injury, *n* = 158	Injury, *n* = 59	Unadjusted OR (95% CI)	*P* value
Morphological changes				
ȃT-wave inversion	15	31	5.535 (2.7894–10.981)	<0.001
ȃST segment depression	11	22	5.356 (2.448–11.72)	<0.001
ȃQTc prolongation	12	17	3.794 (1.709–8.42)	0.001
ȃFlat T-wave	22	4	0.487 (0.161–1.472)	0.2
ȃU-wave	8	7	2.343 (0.813–6.747)	0.11
ȃHyperacute T-wave	3	2	1.785 (0.291–10.953)	0.53
Arrythmias				
ȃAtrial fibrillation	45	24	1.4282 (0.8–2.55)	0.23
ȃAV block I degree	19	5	0.7047 (0.25–1.97)	0.51
ȃRBBB	12	5	1.11 (0.377–3.303)	0.84
ȃSinus bradycardia	11	4	0.97 (0.298–3.178)	0.96
ȃLAH	5	5	2.68 (0.758–9.585)	0.13
ȃSinus tachycardia	4	4	2.678 (0.648–11.055)	0.17

AV, atrioventricular; LAH, left anterior hemiblock; RBBB, right bundle branch block.

Atrial fibrillation was the most common arrhythmia, occurring in almost one-third of the patients during the first 48 h of hospitalization. Among the patients with myocardial injury atrial fibrillation occurred in 40.6%. However, there was no association between patients with myocardial injury and without injury (*P* = 0.23).

In contrast, acute myocardial injury was associated with new abnormality in regional wall motion of the LV compared with patients with no injury (*P* = 0.044, OR 5.546, 95% CI 1.051–29.267), but there was no association between LV global function evaluated based on ejection fraction.

## Discussion

Troponin elevation after AIS is a common finding, but neither its aetiology nor its mechanism is fully understood, as it may be influenced by several pathological conditions, such as catecholamine release and systemic inflammation, or may occur concomitantly with conditions such as heart failure, renal insufficiency, chronic obstructive pulmonary disease, pulmonary embolism, sepsis, and atrial fibrillation. Nonetheless, the prognostic significance of troponin elevation is becoming clear.^15–17^

To the best of our knowledge, this is the first study to examine acute and chronic myocardial injury in patients after AIS, its association with stroke severity, and the prognosis of these patients. The prevalence of myocardial injury in our AIS patients was 27.2%, which is in accordance with previous studies.^17–19^ Chronic myocardial injury was diagnosed in 16% and acute myocardial injury was diagnosed in 11% of patients. In both groups, the proportion of females was higher.

The Fourth Universal Definition of Myocardial Infarction includes the hs-cTnI level and distinguishes between myocardial infarction and myocardial injury. Acute myocardial injury is defined as an hs-cTnI level above the 99th percentile URL and an increase and/or decrease in the hs-cTnI level > 20%. In the absence of the latter, the diagnosis is chronic myocardial injury.^20^ In our study, this definition was used to define prognostic significance, although our results showed that both acute and chronic myocardial injury are associated with stroke severity, poor prognosis, and death. The association with death and stroke severity was stronger in patients with acute myocardial injury. In all patients with myocardial injury, but particularly those with acute injury, the risks for a poor prognosis and all-cause mortality should be determined. The levels of prognostic biomarkers should be measured to optimise care and to determine the need for long-term neurological and cardiologic therapy. Although the aetiology, such as silent myocardial injury, abnormal catecholamine release, induced apoptosis, and systemic inflammation,^21–23^ is not easily determined, even basic information regarding mortality risk can improve patient management and reduce the risk for complications. Close, daily cooperation between the cardiologist and the neurology clinic is therefore recommended. In a stroke unit, the team should include a cardiologist charged with identifying cardiovascular conditions that may have contributed to the stroke and then treating those conditions to reduce the risk of death.

This study had several limitations. First, it was a single-centre study, and the included patients were heterogeneous regarding the type of reperfusion therapy. The length of ECG monitoring depended on the clinical condition of the patients, and drug treatment as a possible confounder was not investigated, nor was the possible influence of the location of the brain lesion on the occurrence of myocardial damage.

In conclusion, an elevated hs-cTnI level is a common finding in AIS patients which could trigger exhaustive diagnostic investigations and consultation. Despite the limitations of our study, its findings extend the current understanding of the implications of an elevated hs-cTnI level in AIS patients. Compared with a single measurement, repeat measurements in the acute phase after ischaemic stroke and an assessment according to standard criteria may provide more accurate information on the type of myocardial injury in AIS patients and better identify patients at increased risk for all-cause mortality and the need for cardiologic consultation. Whether the treatment and long-term observation of patients with an elevated hs-cTnI level can reduce cardiac morbidity and mortality should be the focus of future research.

## Data Availability

The data underlying this article will be shared on reasonable request to the corresponding author.
